# Framework for Laplacian-Level Noninteracting Free-Energy Density Functionals

**DOI:** 10.1021/acs.jpclett.4c01521

**Published:** 2024-08-06

**Authors:** Valentin V. Karasiev, Joshua Hinz, R. M. N. Goshadze

**Affiliations:** Laboratory for Laser Energetics, University of Rochester, 250 East River Road, Rochester, New York 14623-1299 United States

## Abstract

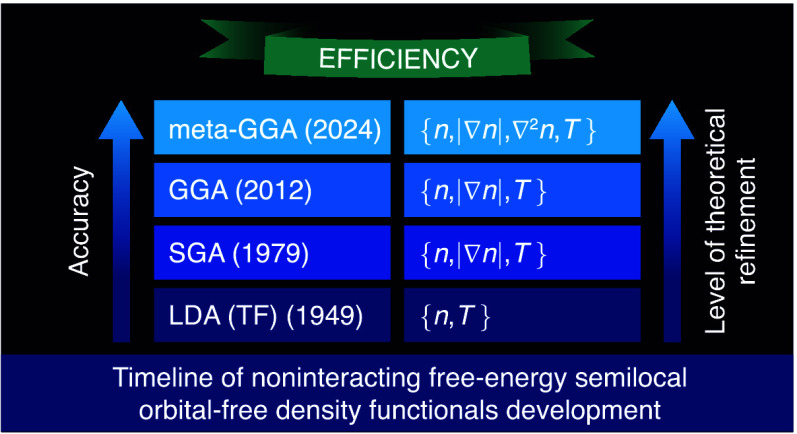

A framework for orbital-free Laplacian-level meta-generalized-gradient approximation (meta-GGA) for the noninteracting free-energy-density functionals based upon analysis of the fourth-order gradient expansion is developed. The framework presented here provides a new tool for developing advanced orbital-free thermal functionals at the meta-GGA level of theory. A nonempirical meta-GGA functional, which in the slowly varying density limit correctly reduces to the finite-temperature fourth-order gradient expansion for the noninteracting free energy, is constructed. Application to warm dense helium demonstrates that the developed meta-GGA functional drastically increases the accuracy of orbital-free-density functional theory simulations at temperatures below 40 eV as compared to the lower Thomas–Fermi and GGA rungs.

Orbital-free (OF) density functional theory (DFT) is a very attractive and sometimes unique alternative to the quantum-mechanical treatment of large systems at elevated temperatures in the warm-dense-matter regime due to its favorable scaling with respect to both the system size and temperature as compared to the standard orbital-based Mermin–Kohn–Sham (MKS) method.^1,2^ The development of noninteracting kinetic energy density functionals for zero-*T* applications began in 1927 when the simplest Thomas–Fermi approximation^3,4^ was proposed. The list of modern noninteracting kinetic energy density functionals consists of tens of semilocal (one-point), nonlocal (two-point) and machine-learnt functionals.^5^ Nonlocal functionals fulfill the homogeneous electron gas (HEG) density response condition and as a consequence provide good accuracy when predicting the bulk properties of metals. However, the applicability of two-point functionals to semiconductors and insulating systems with highly inhomogeneous electron density remains a challenge.^6^ Recently developed semilocal functionals at the meta-generalized gradient approximation (meta-GGA) level of refinement^7,8^ represent state-of-the-art in the orbital-free development at zero-*T*.

At finite-*T* the noninteracting free-energy density functional, consisting of contributions from the noninteracting kinetic energy and noninterating entropy, is the main ingredient of the orbital-free approach. Although the history of the development noninteracting free-energy functionals begun over 70 years ago when Feynman and collaborators developed finite-temperature version of the Thomas–Fermi (TF) theory,^9^ the record of functional development in that field is relatively short. It includes the finite-*T* second-order^10^ and fourth-order^11−13^ gradient expansions (GE4), development of a generalized gradient approximation (GGA) framework,^14^ and a nonlocal two-point functional.^15,16^

With both the rapidly growing interest in high-energy density physics and related experimental accessibility issues, there is the need for the development of reliable and efficient computational methods to predict properties of matter. As warm-dense matter is characterized by elevated temperatures and a wide range of pressures, *ab initio* molecular dynamics approaches (AIMD) require a quantum treatment of the electronic degrees of freedom which can be particularly challenging. For instance, the computational cost of AIMD simulations driven by orbital-based MKS interatomic forces has unfavorable *O*(*N*^3^*T*^3^) scaling with respect to the system size and temperature. However, this can be overcome by OF-DFT which has a computational cost that scales nearly linear with system size, *O*(*N* ln *N*), irrespective of temperature.^17,18^

The simplest TF functional, corresponding to the local density approximation (LDA), is accurate at very high temperatures only. Two successful realizations of GGA-level functionals^19,20^ improve the accuracy of theoretical predictions at intermediate temperatures compared to the TF approximation. A one-parameter tunable OF GGA functional^17^ recently was used to construct a wide-range equation-of-state table for pure deuterium^21^ and for a CHON quaternary compound,^22^ demonstrating the practical utility and importance of orbital-free density functional development for inertial confinement fusion, plasma physics, and planetary science applications.

However, the orbital-free GGA-level functionals usually become reasonably reliable starting at temperatures of a few tens of eV and increase the accuracy as *T* increases. The next-rung meta-GGA functionals are expected to be more accurate at lower temperatures, which will increase the overall reliability of the predictions compared to the LDA- and GGA-level theory.

In this Letter, we address this problem by developing a framework for orbital-free Laplacian-level approximations (meta-GGA’s) for the noninteracting free-energy density and its components (kinetic energy and entropy) based upon analysis of the finite-*T* GE4. This development is the main result of this paper. It opens new perspectives for the construction of advanced meta-GGA-level noninteracting free-energy orbital-free density functionals that are reliable across an extended range of thermodynamic conditions. The orbital-free approach, as advanced in the present work, allows fast and accurate simulations of matter across temperature regimes required for the construction of wide-ranging equations of state, and for predicting viscosity, mutual diffusion coefficients, and dynamic structure factors in regimes when the orbital-based MKS computations become prohibitively expensive due to increased system size and/or elevated temperature.^23−25^ With this work, the finite-*T* orbital-free DFT has reached the same level of theoretical refinement as the mainline ground-state DFT, representing a significant step forward.

Beginning, the fourth-order gradient corrections to the finite-temperature Thomas–Fermi model^9^ have been derived almost simultaneously by Geldart and Sommer^11,12^ and by Bartel et al.^13^ The free-energy density of a system of noninteracting electrons takes the following form
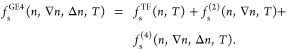
1

The Thomas–Fermi term is given as *f*_s_^TF^(*n*,*T*) = τ_0_^TF^(*n*)κ(*t*) = τ_0_^TF^(*n*)[ξ(*t*) – ζ(*t*)], where , and κ, ξ and ζ are functions of reduced temperature  [where β = 1/(*k*_B_*T*)] and may be represented in the form of explicit fits [see the Supporting Information (SI)^26^ and refs^10,14,27^ for details].

The second-order term (also derived previously by Perrot^10^) is equal to
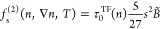
2where  is reduced density gradient and the function *B̃* derived in ref^11^ is equal to the function h̃ used in refs.,^10,14^ which can be expressed as a combination of Fermi–Dirac integrals
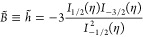
3where *I*_α_ is the Fermi–Dirac integral^13^
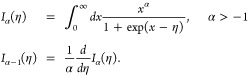
4

Here η = *βμ*, and μ is the local chemical potential defined by density *n*(**r**) through the following relation
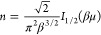
5Due to the strictly increasing behavior of function *I*_1/2_(η), η = *βμ* and all functions of η are functions of reduced temperature *t*. Alternatively (and equivalently), variable *y* ≡ *I*_1/2_(η) = 2/3*t*^3/2^ can be used instead of reduced temperature *t*. In such a way, by elimination of the η variable in eq 3, the function *B̃* becomes a function of *t* (or equivalently a function of *y*).

The fourth-order contribution has three terms
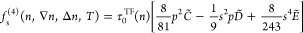
6where *s* is defined below eq 2;  is reduced density Laplacian; and *C̃*, *D̃*, and *Ẽ* are functions of the variable η represented as combinations of Fermi–Dirac integrals [see the SI(26) and eqs 14–16 in ref^27^ for explicit definitions]. Likewise in the case of function *B̃*, the variable η can be eliminated and *C̃* – *Ẽ* quantities become functions of the *t* (or *y*) variable only. The SI(26) gives details of the *B̃*, *C̃*, *D̃*, and *Ẽ* analytical fits developed in ref. .^27^Figure S2 in the SI shows the behavior of functions *B̃*(*t*) – *Ẽ*(*t*). All functions are reduced to 1 in the zero-*T* limit. That means that the finite-*T* gradient expansion defined by eqs 1, 2 and 6 reduces to the Kirzhnits–Hodges zero-*T* gradient expansion for kinetic energy.^28,29^

At this point, we define the meta-GGA (or MGGA) noninteracting free-energy functional form consisting of the noninteracting kinetic energy and noninteracting entropic terms as
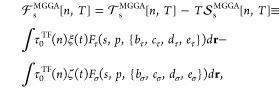
7where, besides the usual *s* and *p* dependence, two sets of variables, {*b*_τ_, *c*_τ_, *d*_τ_, *e*_τ_} and {*b*_σ_, *c*_σ_, *d*_σ_, *e*_σ_} define an explicit *T*-dependence in the kinetic and entropic enhancement factors. These *T*-dependencies, based upon analysis of the finite-*T* fourth-order gradient expansion eqs 1, (2) and (6), are defined in the next few paragraphs.

The definition in eq 7 of  by construction provides the correct scaling of the kinetic and entropic contributions.^30,31^ Due to τ_0_^TF^(*n*) being the only dimensional term in , the kinetic energy and the entropy scale as  and , with *n*_λ_(**r**) = λ^3^*n*(λ**r**).

The fourth-order gradient expansion given by eqs 1, 2 and 6 can be presented in the following factorized form
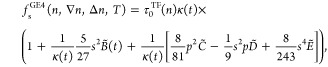
8where the sum of dimensionless terms in parentheses can be interpreted as a single enhancement factor for the fourth-order noninteracting free-energy gradient expansion defined in terms of the dimensionless reduced density gradient and Laplacian variables corresponding to free-energy: , ,  and . However, it becomes clear that all these variables have a pole because the function κ(*t*), which appears in the denominator of each of the above-defined variables, has a zero at *t* ≈ 0.54, see Figure S1 in SI (see also discussion in Sec. V of ref^14^). Therefore, we follow the approach used for the development of the finite-temperature GGA^14^ in terms of the reduced density gradients for the nonnegative kinetic and entropic contributions, respectively. We define fourth-order temperature-dependent dimensionless variables to be used in the kinetic and entropic enhancement factors of meta-GGA functionals. Kinetic and entropic contributions corresponding to the fourth-order term in eq 6 can be evaluated by invoking the standard thermodynamic relation
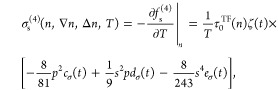
9where *t*-dependent functions, attached to *p*^2^, *s*^2^*p* and *s*^4^ reduced density variables, are defined as
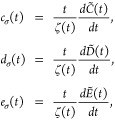
10for the entropy density, and
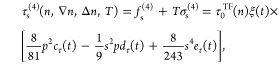
11where
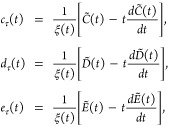
12for the kinetic energy density. Together with the second-order kinetic and entropic temperature dependent functions  and , eqs 10 and 12 form the full set of meta-GGA level functions that describe the temperature dependencies of the 2-nd and 4-th order kinetic and entropic terms, respectively.

Figure 1 shows the behavior of functions *b*_τ_, *c*_τ_, *d*_τ_, and *e*_τ_ and *b*_σ_, *c*_σ_, *d*_σ_, and *e*_σ_. Kinetic *b*_τ_, *c*_τ_, *d*_τ_ and *e*_τ_ variables are non-negative functions of reduced temperature *t* with a zero-*t* limit equal to one, with some structure at intermediate *t* and decaying at large *t*. Entropic functions *b*_σ_, *c*_σ_, *d*_σ_ and *e*_σ_ have the zero-*t* limit varying between ≈0.8 and 4.7, and decay very fast for *t* > 1. Functions *c*_σ_, *d*_σ_ and *e*_σ_ become negative for *t* greater than a few tenths. Entropic contribution to free energy vanishes at *t* = 0 for the second- and fourth-order terms *Tσ*_s_^(2)^ and *Tσ*_s_^(4)^ due to the ζ(*t*) factor in these terms approaching zero very fast for *t* < 0.1, see Figure 1 in ref.^14^

**Figure 1:**
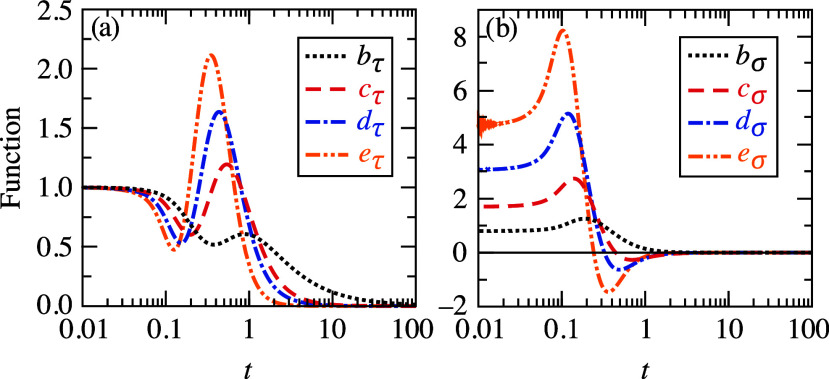
Panel (a): Behavior of kinetic *b*_τ_(*t*), *c*_τ_(*t*), *d*_τ_(*t*), and *e*_τ_(*t*) variables as functions of reduced temperature *t*. Panel (b): Behavior of entropic *b*_σ_(*t*), *c*_σ_(*t*), *d*_σ_(*t*) and *e*_σ_(*t*) variables as functions of reduced temperature *t*.

Two terms in eq 7,  and , have the exact interpretation as the kinetic energy and entropic contributions only if this interpretation is enforced by invoking the thermodynamic relation
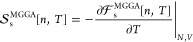
13

After some rearrangement and interchange of integration and partial derivative evaluation one derives a differential equation, which gives exact relation between the *F*_τ_ and *F*_σ_ enhancement factors
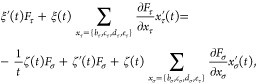
14where primes denote derivatives with respect to argument, i.e., with respect to *t*.

Instead of solving the differential eq 14 (finding unknown *F*_σ_ corresponding to a given *F*_τ_), one may use an approximate relation between kinetic and entropic enhancement factors based on the exact relation between the two gradient expansion enhancement factors. The gradient expansion free energy eq 1 can be presented in the MGGA form with enhancement factors defined as
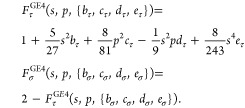
15

The second equation in eq 15 implies that the approximate relation between *F*_τ_ and *F*_σ_ meta-GGA enhancement factors could be used as in the finite-*T* GGA case^14^
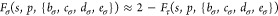
16eqs 10, 12–14, and 16 define the framework for constructing the Laplacian-level noninteracting free-energy functionals and represent the main result of the present work. For the construction of advanced meta-GGA noninteracting free-energy density functionals, one needs to develop a new or use an existing ground-state kinetic energy enhancement factor, *F*_t_(*s*, *p*). Variables defined by eqs 10 and 12 will be used to incorporate temperature dependencies in such a ground-state enhancement factor to construct its thermal version. Eventually, the noninteracting free-energy functional will be given by eq 7 and by an approximate relation between the entropic and kinetic enhancement factor eq 16. Instead of approximating eq 16, one may solve differential eq 14 to find *F*_σ_. Entropic and kinetic contributions can be found by eq 13 and  respectively.

Within the developed framework, we propose a (d)ouble-(e)exponent *s*-dependent kinetic-energy enhancement factor with additional linear fourth-order (L)aplacian dependent terms (DEL)
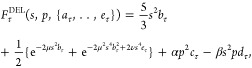
17with μ = 40/27, ν = 8/243, α = 8/81, and β = 1/9. The first term in eq 17 corresponds to the VW contribution. The Pauli term is represented by the double-exponent term in braces augmented by the fourth-order Laplacian dependent contributions (eq 15). The double-exponent term is required to reproduce the second- and fourth-order *s*-dependent terms of the gradient expansion eq 15 in the small-*s* limit; simultaneously, it regularizes the potential large-*s* divergencies. The entropic enhancement factor is defined as in eq 16, i.e., *F*_σ_^DEL^ = 2-*F*_τ_^DEL^. Equation 17 recovers the fourth-order finite-*T* gradient expansion eq 15 in the slowly varying density limit.

The framework described above makes a finite-*T* extension of existing ground-state meta-GGA kinetic energy functionals straightforward in many cases. First, we notice that a convenient partition of the ground-state kinetic-energy functional on the von Weizsäker (VW) and the remainder, referred to as the Pauli term (which for the ground-state enhancement factor is *F*_t_ = (5/3)*s*^2^ + ...) at finite-*T*, takes the following form: *F*_τ_ = (5/3)*s*^2^*b*_τ_ + ... Consider for example the Pauli–Gaussian second order plus Laplacian term (PGSLr) meta-GGA kinetic energy introduced in ref^8^ defined by the following temperature-independent enhancement factor . After recognizing the relation of terms to the fourth-order gradient expansion the finite-*T* extension of the PGSLr meta-GGA has the following temperature-dependent kinetic and entropic enhancement factors: , and *F*_σ_^PGSLr^(*s*,*p*,{*a*_σ_,..,*e*_σ_})=2-*F*_τ_^PGSLr^(*s*,*p*,{*a*_σ_,..,*e*_σ_}), respectively.

The accuracy of the developed meta-GGA/DEL functional with respect to the lower TF and GGA rungs is investigated by performing AIMD simulations for dense D (along one isochore), He (along two isochores), dense LiD along one isochore, and Al at melting and warm dense conditions. All orbital-free and some part of orbital-based AIMD simulations were performed with the PROFESS@QUANTUM-ESPRESSO package.^18,32−34^ using an Andersen or Berendsen thermostat^35,36^ and all-electron local pseudopotentials^22,37^ (LPPs).

The orbital-based MKS approach, reliable for a wide range of applications, provides an exact treatment for noninteracting free-energy. This makes MKS the ideal reference data for the OF-DFT approach to assess the accuracy of the approximate noninteracting free-energy functionals, given that the exchange-correlation functional employed in both calculations is the same, and that used pseudopotentials are accurate, preventing any error cancellations.

The reference MKS simulations for He, LiD and Al were performed using the plane wave Vienna *ab initio* simulation package (VASP).^38^ MKS simulations were done at the Γ-point only or employed the Baldereschi mena-value *k*-point.^39^ Further computational details are provided in SI, Sec. III. MKS calculations at high temperatures are not feasible due to very high computational cost. In such cases, high-quality path-integral Monte Carlo (PIMC) data^40,42^ have been used as a reference. The PIMC approach, developed for high-temperature applications, does not require any approximations of the XC energy. Depending on the magnitude of the XC thermal effects, DFT calculations require the use of a thermal XC functional to be compatible with the PIMC reference. Two thermal XC functionals, KDT16^43^ and TSCANL,^44^ employed in our calculations for He and LiD, provide an excellent description of thermal effects (see details in^44^), making our DFT calculations compatible with the PIMC reference. However, in our calculations for D and Al, we had to use the ground-state LDA XC, the same as in ref.,^16^ to provide fair comparisons between our meta-GGA/DEL and nonlocal^16^ orbital-free functionals.

Comparison between the finite-*T* meta-GGA/DEL/PGSLr/GE4, GGA/LKT,^20^ TF,^9^ two-point nonlocal^16^ orbital-free functionals and the MKS and PIMC^40^ reference data is shown in Figure 2. Our meta-GGA/DEL and two-point nonlocal free-energy functionals demonstrate very similar performance; the relative deviation for these two functionals with respect to either the MKS or PIMC results never exceed 2% across the entire range of temperature. These discrepancies essentially lie within the error bars of a typical AIMD simulation when the combined statistical and convergence errors with respect to the system size, energy cutoff, etc. are accounted for, which are around 2%. We estimate that the difference between our MKS simulations and the PIMC data does not exceed 1% providing accurate references. Both the meta-GGA/DEL and nonlocal free-energy functionals demonstrate similar trends with variation of the material density (see ref^16^): accuracy decreases with decrease of the density, and it remains at the similar level for denser systems.

**Figure 2:**
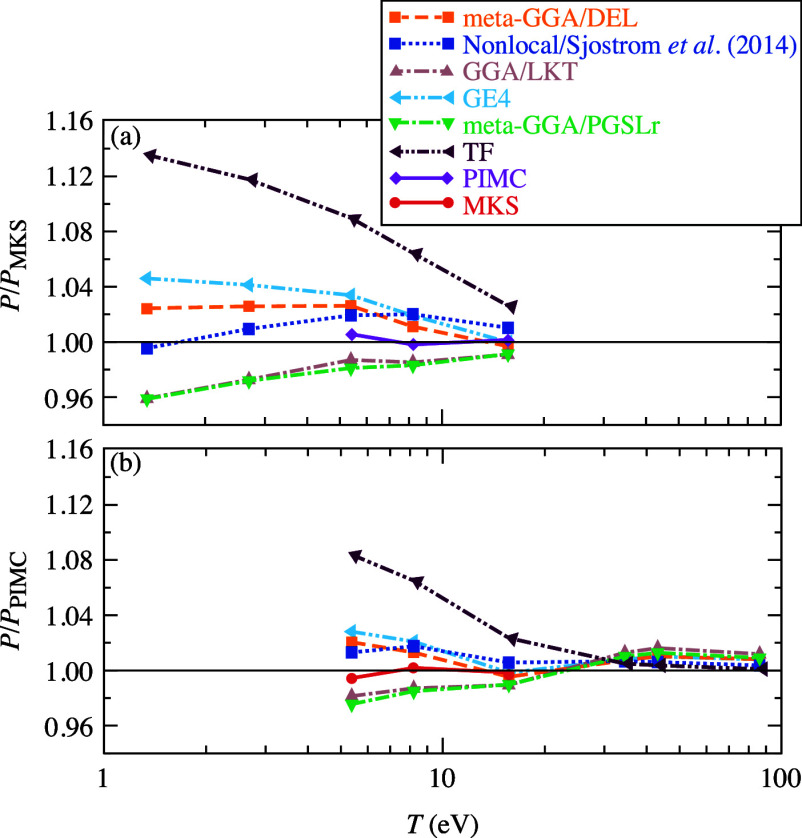
Relative pressure with respect to MKS (a) and PIMC (b) values^40^ in respective temperature ranges for D at 4.04819 g/cm^3^. All OF-DFT and MKS calculations use the LDA ground-state^41^ XC. The relative pressures from calculations with GGA/LKT,^20^ the fourth-order gradient expansion (GE4) and meta-GGA/PGSLr orbital-free functionals are included additionally.

The TF error slowly decays from 14% to 4% with temperature increase between 1.35 and 15.65 eV, eventually reaching the level below 2% at *T* above 30 eV. The PGSLr and GE4 meta-GGA, and LKT GGA have the similar magnitude of relative error which is slightly above 4% at 1.35 eV and decreases to the level around 2% and below at *T* above 8 eV.

Dense deuterium at temperatures of 1 eV and above can be characterized as a conducting and modestly inhomogeneous (with respect to the electron density) system. The existing nonlocal two-point functional was designed to be accurate for conducting weakly inhomogeneous systems such that it provides good accuracy. The simplest TF approximation does not take into account any inhomogeneity effects and becomes accurate only in the high-*T* limit (i.e., in the HEG limit). The next rung functional, GGA/LKT, takes into account the combined inhomogeneity and thermal effects at the GGA level, and the accuracy of predictions is improved. The fourth-order gradient expansion functional (meta-GGA/GE4) is accurate only in the limit of a small reduced density gradient and reduced Laplacian values. The developed meta-GGA/DEL functional provides the best accuracy (same level as the nonlocal one) because this functional not only reduces to the fourth-order gradient expansion in the weakly inhomogeneous limit (as opposite to the meta-GGA/PGSLr functional, such that meta-GGA/PGSLr is a less accurate functional) but also provides a generalization at the meta-GGA level improving accuracy in the ranges beyond the near-homogeneous limit. Due to the lack of improvement shown by PGSLr and GE4 meta-GGA compared to the lower-rung GGA functional, these two functionals are excluded from the tests for other systems discussed below.

Now we switch to dense He, an insulating system with a highly inhomogeneous electron density. Figures 3 and S5 in the SI (ρ = 0.3870 and 0.6690 g/cm^3^ respectively) compare the TF, Luo–Karasiev–Trickey (LKT) GGA, and our eq 17 meta-GGA total pressures relative to the high-quality path-integral PIMC data data^42^ combined with KS data at *T* below 10 eV. PIMC simulations take into account thermal exchange-correlation (XC) effects. To make our DFT results thermodynamically consistent with the PIMC data, all orbital-free and Kohn–Sham AIMD simulations were performed with the thermal TSCANL meta-GGA level XC functional, which demonstrated unprecedented accuracy between a few tenths and 1% with respect to the PIMC data for warm dense helium.^44^ Thomas–Fermi overestimates pressure by 55% for both material densities at *T* = 7 eV, and then the error decreases with an increase of temperature and becomes 2% or less at *T* about 40 eV and above. The thermal GGA/LKT functional at *T* = 3.4 eV underestimates pressure by 35% and 43% for ρ = 0.3870 g/cm^3^ and 0.6690 g/cm^3^, respectively. The error decreases to 2% or less at *T* around 10 eV, but it starts to increase at *T* around 20 eV, overestimating pressure by 4% or so. The error decreases with further increase of temperature and becomes 2% or less at *T* above 40 eV and above 50 eV for ρ = 0.3870 g/cm^3^ and 0.6690 g/cm^3^ respectively. Meta-GGA orbital-free simulations provide much more accurate results as compared to GGA in the temperature range between 3.4 and 7 eV. The error decreases to 2% or less for *T* ≥ 7 eV.

**Figure 3:**
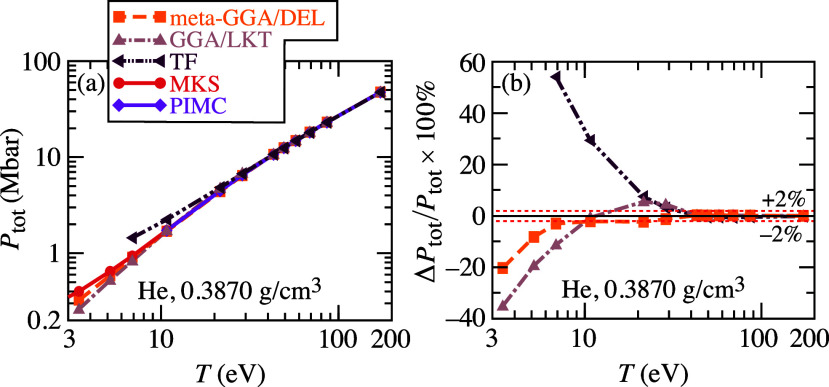
(a) The total pressure from the orbital-free DFT AIMD simulations of warm dense He at ρ = 0.3870 g/cm^3^ (*r*_s_ = 2.4 bohr) with meta-GGA/DEL [eq 17] GGA/LKT and TF functionals, and the the reference MKS DFT DFT and PIMC data.^42^ (b) The relative error of total pressure from OF-DFT simulations with respect to the combined MKS and PIMC data.

Another example includes calculations for warm-dense LiD (along the ρ = 3.4 g/cm^3^ isochore) using all electron pseudopotentials. Active treatment of 1s-core electrons is a challenging task for an orbital-free approach. The GGA/LKT functional underestimates pressure by 15% at 13 eV (see Figure S8), the relative error gradually decreases to the level 2% and below at 28 eV and above. The pressure error of the meta-GGA/DEL free-energy is lower almost by a factor of 2; the error becomes 2% and below at 23 eV and above demonstrating again significant improvement as compared to the GGA-level free-energy functional.

The developed meta-GGA/DEL functional demonstrates a significant improvement in the accuracy for the considered dense He and LiD systems because it provides a better description of the combined inhomogeneity and thermal effects as compared to the lower rung TF and GGA/LKT functionals.

The last example includes calculation of the radial distribution function (RDF) for Al at near melting condition (ρ = 2.349 g/cm^3^, *T* = 0.088 eV) and warm-dense (ρ = 2.7 g/cm^3^, *T* = 5 eV) conditions shown in Figure S10. Meta-GGA/DEL, GGA/LKT functionals and the MKS reference (all combined with the Karasiev–Dufty–Trickey (KDT16)^43^ thermal XC, and orbital-free simulations performed with the Heine-Abarenkov^45,46^ LPP) results are in good agreement at warm-dense conditions. At the lower temperature, our functional demonstrates great improvement compared to the GGA/LKT predictions, providing a good agreement with the MKS reference except for a small discrepancy between the magnitude and location of the two peaks. The nonlocal free-energy functional provides very good agreement with the reference MKS results at both temperatures (see Figure 5 in ref^16^).

Figures S3 and S4 in the SI compare the OF-DFT total pressures, internal energy differences, and total internal energies to the reference MKS and PIMC data for the dense deuterium discussed dense deuterium. The internal and free energy differences for warm dense He (along two isochores) and LiD (one isochore) from the OF-DFT and MKS AIMD simulations are shown in Figures S6, S7 and S10 (panels (a) and (b)) in the SI. Panels (c) in these figures compares total internal energies. In all cases, we observe that the new meta-GGA/DEL orbital-free functional provides significant improvements of accuracy with respect to the MKS reference, as compared to the lower rung LDA/TF and GGA/LKT approximations. The total pressure, internal energy differences and total internal energies for D, He, and LiD are tabulated in Tables S5–S8, see additional discussion in Sec. IV in the SI.

In summary, we have presented a framework for Laplacian level noninteracting free-energy orbital-free density functionals. Dimensionless reduced density gradient and Laplacian variables for the kinetic and entropic components, defined upon analysis of the finite-*T* fourth-order gradient expansion, are employed in the construction of respective meta-GGA enhancement factors *F*_τ_ and *F*_σ_. The developed framework, by construction, incorporates explicit temperature dependencies physically motivated by the finite-*T* fourth-order gradient expansion and ensures the correct scaling of the kinetic and entropic terms. The meta-GGA framework provides a new tool for developing advanced orbital-free thermal functionals, thus representing the main result of the work. As an example, within the developed framework, we presented a simple double-exponent functional with Laplaciant-dependent terms. This functional, in the slowly varying density limit, recovers the fourth-order finite-*T* gradient expansion. AIMD simulations of warm-dense helium demonstrated a drastic increase of the accuracy of orbital-free functionals at temperatures below 40 eV. The lowest temperature when OFDFT predictions are accurate within 2% for dense He is pushed down from 40 eV to a level below 10 eV. Similar improvements of our meta-GGA/DEL with respect to the lower GGA-rung free-energy functional are also observed for dense D, LiD, and Al. The orbital-free approach, as advanced in the present work, allows fast and accurate simulations of matter across temperature regimes required for the construction of wide-range equation of states, predicting viscosity, mutual diffusion coefficients, and dynamic structure factors in regimes when the orbital-based MKS computations become prohibitively expensive due to increased system size and/or elevated temperature.^23−25^

Nonlocal two-point free-energy functionals are tied to the HEG density response (Lindhard) function, which is a justified and reasonable approximation for (semi) metallic systems with weakly inhomogeneous electron density. It is not surprising that existing nonlocal functionals provide excellent accuracy for metallic systems such as warm dense D and Al. However, it is expected that such nonlocal functionals with an enforced homogeneous gas response will not perform well when applied to insulators and systems with highly inhomogeneous electron densities, such as He and LiD, treated with hard all-electron pseudopotentials when the HEG approximation fails.

## Supporting Information Available

Additional theoretical and computational details, additional results (PDF)
