# Supplementary material for: Framework for Laplacian-Level Noninteracting Free-Energy Density Functionals
Source: J Phys Chem Lett. 2024 Aug 6;15(32):8272–9. doi: 10.1021/acs.jpclett.4c01521 (PMC11331511; doi:10.1021/acs.jpclett.4c01521)
Supplement: Supplementary file 1 — jz4c01521_si_001.pdf [file jz4c01521_si_001.pdf]

Supporting Information - "Framework for Laplacian-Level Noninteracting Free-Energy Density Functionals"

Valentin V. Karasiev,* Joshua Hinz, and R. M. N. Goshadze
Laboratory for Laser Energetics, University of Rochester,
250 East River Road, Rochester, New York 14623-1299 USA

I. DEFINITIONS AND ANALYTIC FITS TO ξ AND ζ AS FUNCTIONS OF THE REDUCED TEMPERATURE t

The Thomas-Fermi noninteracting free-energy density can be presented in the following factorized form[1]

$$f_{\rm s}^{\rm TF}(n,T) \;=\; \tau_0^{\rm TF}(n)\kappa(\beta\mu)\,, \tag{S1}$$

where

$$\kappa(\beta\mu) \;=\; \frac{5}{2}\left(\frac{3}{2}I_{1/2}(\beta\mu)\right)^{-5/3}\times \Big[-\frac{2}{3}I_{3/2}(\beta\mu)+\beta\mu I_{1/2}(\beta\mu)\Big]\,. \tag{S2}$$

with $\beta = 1/(k_{\rm B}T)$, $\tau_0^{\rm TF}(n) = 0.3(3\pi^2)^{2/3}n^{5/3}$, and μ is the local chemical potential defined by the electron density n

$$n = -\frac{1}{V}\frac{\partial\Omega}{\partial\mu}\Big|_{T,V} = \frac{\sqrt{2}}{\pi^2\beta^{3/2}}I_{1/2}(\beta\mu)\,, \tag{S3}$$

where Ω is the grand canonical potential of the noninteracting uniform electron gas of density n in a volume V. Equation (S3) gives

$$I_{1/2}(\beta\mu) = \frac{n\pi^2\beta^{3/2}}{\sqrt{2}} = \frac{2}{3t^{3/2}}\,. \tag{S4}$$

where t is the reduced temperature

$$t = T/T_{\rm F} = \frac{2}{\beta[3\pi^2 n]^{2/3}}\,. \tag{S5}$$

Because $I_{1/2}(x)$ is strictly increasing with x, $(\beta\mu)$ is a function of t, hence all functions of $(\beta\mu)$ are functions of t. Variable $(\beta\mu)$ can be eliminated in favor of t in Eq. (S2). That was done numerically, and $\kappa(t)$ was presented analytically as an adapted form of Perrot's free energy fit[2]. The fit for function $\kappa(t)$ is split into regions $t \le t_0$ and $t > t_0$, where $t_0 = 4(2/3\pi^2)^{1/3}/3 = 0.543010717965$ (see further details in Ref.[1]).

For $t \le t_0$

$$\kappa(t) \;=\; 1 - 4.1123335167t^2 + 1.9957322255t^4 + 14.83844536t^6 - 178.4789624t^8 +992.5850212t^{10} - 3126.965212t^{12} + 5296.225924t^{14} - 3742.224547t^{16}\,. \tag{S6}$$

and for $t > t_0$,

$$\kappa(t) \;=\; -2.5t\ln(t) - 2.141088549t + 0.2210798602t^{-0.5} + 0.7916274395\times10^{-3}t^{-2} -0.4351943569\times10^{-2}t^{-3.5} + 0.4188256879\times10^{-2}t^{-5} - 0.2144912720\times10^{-2}t^{-6.5} +0.5590314373\times10^{-3}t^{-8} - 0.5824689694\times10^{-4}t^{-9.5}\,, \tag{S7}$$

The entropy and kinetic energy densities can be found by invoking the standard thermodynamic relation

$$\sigma_{\rm s}^{\rm TF}(n,T) \;=\; -\frac{\partial f_{\rm s}^{\rm TF}(n,T)}{\partial T}\Big|_n \equiv \frac{1}{T}\tau_0^{\rm TF}(n)\zeta(t)\,, \tag{S8}$$

$$\zeta(t) \;=\; -t\frac{d\kappa(t)}{dt}\,, \tag{S9}$$

and

$$\tau_{\rm s}^{\rm TF}(n,T) \;=\; f_{\rm s}^{\rm TF}(n,T) + T\sigma_{\rm s}^{\rm TF}(n,T) \equiv \tau_0^{\rm TF}(n)\xi(t)\,, \tag{S10}$$

$$\xi(t) \;=\; \kappa(t) - t\frac{d\kappa(t)}{dt}\,, \tag{S11}$$

$$\kappa(t) \;=\; \xi(t) - \zeta(t)\,. \tag{S12}$$

The functions $\zeta(t)$ and $\xi(t)$ may be calculated using relations with $\kappa(t)$ given in Eqs. (S9), (S12), and the analytic fit defined by Eqs. (S6) and (S7). Functions κ, ξ, and ζ are shown in Fig. S1.

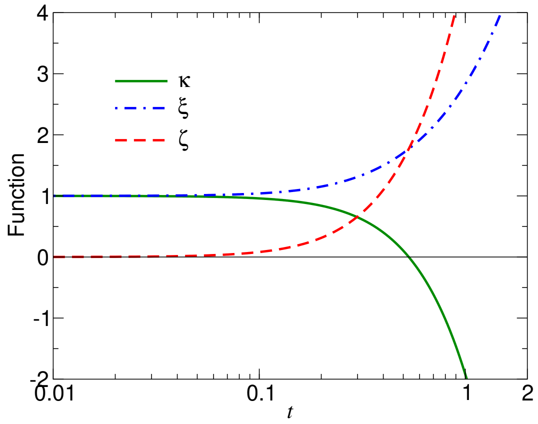

FIG. S1: Behavior of functions $\kappa(t)$, $\xi(t)$ and $\zeta(t)$.

II. DEFINITIONS AND ANALYTIC FITS TO $\widetilde{B}$, $\widetilde{C}$, $\widetilde{D}$, $\widetilde{E}$ AS FUNCTIONS OF THE REDUCED TEMPERATURE t

Functions $\widetilde{B}$, $\widetilde{C}$, $\widetilde{D}$, $\widetilde{E}$ have the following definitions as Fermi-Dirac integral combinations (see Refs.[1–6] for further details):

$$\widetilde{B}(\eta) = -3\frac{I_{1/2}(\eta)I_{-3/2}(\eta)}{I^2_{-1/2}(\eta)}\,, \tag{S13}$$

$$\widetilde{C}(\eta) = \frac{5\times 3^{11/3}}{2^{11/3}} I^{5/3}_{1/2}(\eta)\left[\frac{1}{9}\frac{I^2_{-3/2}(\eta)}{I^3_{-1/2}(\eta)} - \frac{1}{5}\frac{I_{-5/2}(\eta)}{I^2_{-1/2}(\eta)}\right], \tag{S14}$$

$$\widetilde{D}(\eta) = \frac{5\times 2^{1/3}}{3^{1/3}} I^{8/3}_{1/2}(\eta)\left[-3\frac{I_{-7/2}(\eta)}{I^3_{-1/2}(\eta)} + \frac{33}{10}\frac{I_{-3/2}(\eta)I_{-5/2}(\eta)}{I^4_{-1/2}(\eta)} - \frac{I^3_{-3/2}(\eta)}{I^5_{-1/2}(\eta)}\right], \tag{S15}$$

and

$$\widetilde{E}(\eta) = \frac{5\times 3^{14/3}}{2^{2/3}} I^{11/3}_{1/2}(\eta)\left[-\frac{7}{96}\frac{I_{-9/2}(\eta)}{I^4_{-1/2}(\eta)} - \frac{1}{15}\frac{I^2_{-3/2}(\eta)I_{-5/2}(\eta)}{I^6_{-1/2}(\eta)} + \frac{1}{72}\frac{I^4_{-3/2}(\eta)}{I^7_{-1/2}(\eta)} + \frac{1}{12}\frac{I_{-3/2}(\eta)I_{-7/2}(\eta)}{I^5_{-1/2}(\eta)} + \frac{1}{32}\frac{I^2_{-5/2}(\eta)}{I^5_{-1/2}(\eta)}\right], \tag{S16}$$

where $\eta = \beta\mu$.

Smooth analytical representation of $\widetilde{B}$ as a function of variable $y \equiv I_{1/2}(\eta) = 2/3t^{3/2}$ is given by a Padé approximant of order [24,24] with respect to the variable $y^{1/3}$:

$$\widetilde{B}(y) = \frac{\sum_{i=0}^{8} a_i y^i}{1+\sum_{i=1}^{12} b_i u^i}\,, \tag{S17}$$

where $u = y^{2/3}$. For $\widetilde{C}$, $\widetilde{D}$, and $\widetilde{E}$ the fitting fanctions are of the form:

$$R(y) = \frac{a_{2.5}u^{5/2} + \sum_{i=1}^{12} a_i u^i}{1+\sum_{i=1}^{6} b_i v^i}\,, \tag{S18}$$

where $v = y^{4/3}$. Eq. (S18) also has the form of a [24,24] Padé approximant with respect to the variable $y^{1/3}$.

The coefficients of the analytic fits to $\widetilde{B}$, $\widetilde{C}$, $\widetilde{D}$, $\widetilde{E}$ as functions of the variable y determined in Ref.[3] are given in Tables S1-S4. A simple variable replacement $y = 2/3t^{3/2}$ leads to definition of $\widetilde{B}$, $\widetilde{C}$, $\widetilde{D}$, $\widetilde{E}$ as functions of the reduced temperature t.

III. COMPUTATIONAL DETAILS

All orbital-free and most of orbital-based Mermin-Kohn-Sham density functional theory simulations (OF-DFT and MKS-DFT respectively) were performed with the PROFESS@QUANTUM-ESPRESSO package[7–10] using an Andersen[11] or Berendsen thermostat[12]. This packages uses the same molecular dynamics engine and allows employment of the same local pseudo-potential (LPP) in both the OF and MKS methods. Part of the reference MKS simulations were performed using the plane wave Vienna *ab initio* simulation package (VASP)[13]. Accuracy and transferability of our hard all-electron LPPs for D/H, He and Li to warm-dense conditions was tested against

custom developed hard all-electron projector augmented wave (PAW) data sets by comparison between the LPP and PAW Mermin-Kohn-Sham single-point (static) calculations. These comparisons are out of scope of this work and will be reported somewhere else.

A. Deuterium

The LPP for hydrogen/deuterium described and tested in Ref.[1] has been used in both the OF and MSK simulations with 128 deuterium atoms simulated for about 6500 MD steps after thermal equilibration. The MD time step, having the initial value between 0.1 and 0.3 fs at 1eV, was varying depending on temperature as $\sim T^{-1/2}$, and on material density as $\sim \rho^{-1/3}$. We found that results are stable with respect to the initial value of the MD time step. The numeric grid size was 64^3 for the orbital-free simulations. For the MKS ones the plane wave energy cutoff, $E_{\rm cut}$, was set to 100 ry and the calculations were done at the Γ-point only. All simulations for D use the local density approximation (LDA) exchange-correlation (XC) functional[14] to provide consistent comparisons with the two-point non-local free-energy functional data[15].

B. Helium

Orbital-free simulations for dense He were performed with the Hartwigsen-Goedecker-Hutter (HGH) dual-space Gaussian LPP[16]. The potential, defined by Eq. (1) in Ref.[16] was generated using the Perdew-Burke-Ernzerhof (PBE) GGA for XC. Simulations were performed for 64 or 32 atoms depending on the material density and temperature conditions, and the numerical grid size of 128^3. The length of MD runs was between 5000 and 6500 MD steps after thermalization. The reference MKS calculations were performed with Vasp using the standard PBE PAW, the plane-energy cutoff of 1 keV, and the Baldereschi mean value k-point[17]. The thermal TSCANL meta-GGA level XC functional[18] was used in both the orbital-free and MKS Vasp simulations. Additional comparisons between the MKS calculations using the above described HGH LPP performed with Profess@Quantum-Espresso ($E_{\rm cut} = 300$ ry) and PAW Vasp results have been performed.

C. Lithium deuteride

Orbital-free simulations for LiD along $\rho = 3.4$ g/cm^3 isochore and temperature range between 13 eV (150 kK) and 34 eV (400 kK) used all-electron PBE HGH local pseudopotentials and the numeric grid size of 128^3 with 32 Li and 32 D atoms. The thermal KDT16 GGA level XC functional[21] was used in both the orbital-free and MKS Vasp simulations.

MKS simulations used a 3-electron PBE PAW data set, $E_{\rm cut} = 1400$ eV, and employed the Baldereschi mean-value k-point with the same number of atoms, except for T between 26 and 34 eV when the system size was decreased by a factor of two. The time step varied from 32 as to 21 as according to scaling shown in Sec. III A, and simulations were performed for 3000 MD steps after equilibration. Additional comparisons between the MKS calculations using the above described HGH LPP performed with Profess@Quantum-Espresso ($E_{\rm cut} = 300$ ry) and PAW Vasp results have been performed

D. Aluminum

Our orbital-free DFT simulations for 108 Al atoms used the Heine-Abarenkov LPP[19,20], and the 128^3 numeric grid. The MKS reference calculations were performed with Vasp for the same system size using a 3-electron PBE PAW data set, $E_{\rm cut} = 500$ eV, and the Baldereschi mean value k-point. The length of all MD runs was between 5000 and 6500 MD steps after thermalization. All simulations were performed with the Karasiev-Dufty-Trickey (KDT16) thermal GGA XC[21].

E. Implementation in Profess@Quantum-Espresso

Laplacian-level non-interacting free-energy density functionals (DEL, PRSLr and GE4) have been implemented in the PROFESS@Quantum-Espresso large-scale computational package[7]. Implementation followed numerical technique described in detail in Refs.[7–10]. All derivatives, reduced density gradients and reduced Laplacians are evaluated in reciprocal space using fast Fourier transforms (FFTs) leading to nearly-linear $O(N \ln N)$ scaling with respect to the effective system size, and a flat scaling with some decrease of the computational cost with increase of temperature.

Computational cost of the Laplacian-level functionals usually is higher by a factor about four as compared to the lower GGA-rung functionals. This is a very similar increase of computational time as demonstrated by the GGA functionals with respect to the simplest Thomas-Fermi local approximation (see Figs. 3 and 4 in Ref.[7]). However, given that the orbital-based MKS scheme has an unfavorable scaling with respect to the system size and temperature as $O(N^3T^3)$, the near-linear scaling of the orbital-free approach, even with an additional prefactor in the computational cost, makes the Laplacian-level functionals much faster for large systems and/or at elevated temperatures, as compared to the orbital-based approach.

It is expected that Laplacian-level functionals might have some instability or numerical issues. However, the meta-GGA level functionals developed in the present work and applied to a set of described systems did not reveal any issues as compared to the thermal GGA-level func-

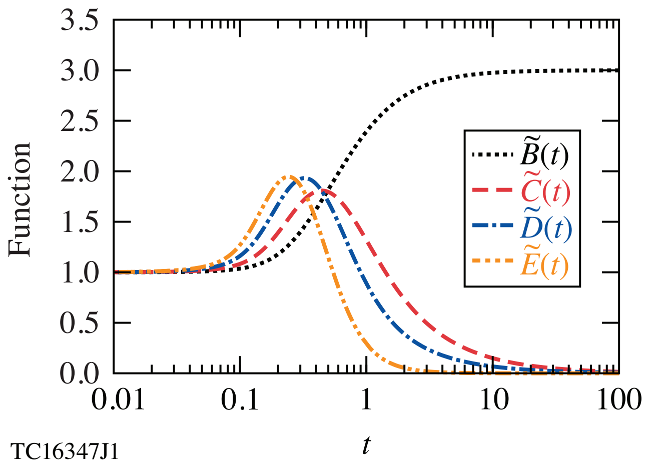

FIG. S2: Behavior of functions $\widetilde{B}(t) - \widetilde{E}(t)$ defined by Eqs. (S13)-(S16).

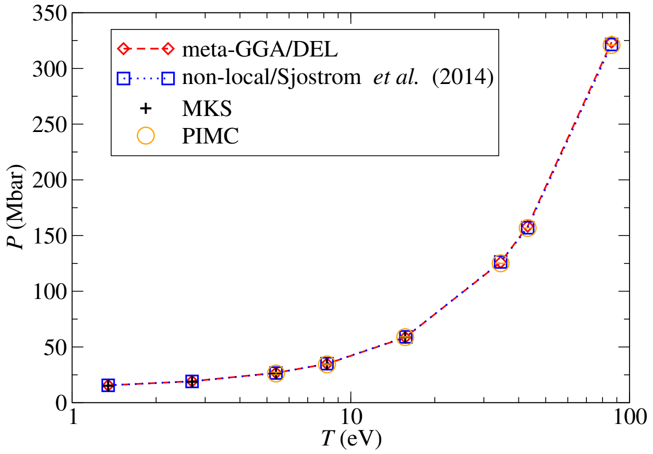

FIG. S3: The total pressure as a function of temperature from the DFT AIMD simulations of warm dense D at ρ = 4.04819 g/cm^3 (r_s = 1.1 bohr) with meta-GGA/DEL [Eq. (15)] and nonlocal (two-point) [Ref.[15]] orbital-free functionals, and the reference Mermin–Kohn–Sham (MKS) DFT and PIMC[22] data. All OF-DFT and MKS calculations use the local density approximation (LDA) ground-state XC[14].

tionals, except that Laplacian-level functionals, depending on state-conditions, may require a larger number of self-consistent field (SCF) iterations to converge to the required accuracy. One issue, relevant to both the GGA and meta-GGA level functionals, is the initial guess for the electron density. For system with highly inhomogeneous electron density al low-T (e.g. dense He), the SCF loop may not converge if started from the homogeneous electron density initial guess (which is the default initial guess in the ground-state OF-DFT implementation). A start from pre-calculated density has been implemented in the PROFESS@QUANTUM-ESPRESSO package. The initial guess might be calculated for a given ionic arrangement (a single-point calculation), but using a higher electron temperature. Once the electron density is converged, it is used as the initial guess in the AIMD simulations with the GGA and meta-GGA level orbital-free functionals.

IV. ADDITIONAL RESULTS

Figure S2 shows the behavior of functions $\widetilde{B}(t) - \widetilde{E}(t)$ defined by Eqs. (S13)-(S16) and discussed in the main text. All functions reduce to unity in the zero-T limit. Functions $\widetilde{C}(t) - \widetilde{E}(t)$ decay and asymptotically approach zero at high temperatures.

Figure S3 shows additional meta-GGA/DEL [Eq. (15)] and non-local (two-point)[15] orbital-free results for dense D. The total pressure as a function of temperature for these two functionals is in perfect agreement with respect to the MKS and PIMC reference. Figures S4 (a) and (b) compare the internal energy differences from the orbital-free and PIMC/MKS reference calculations. All energy differences are calculated along the $\rho = 4.04819$ g/cm^3 isochore w.r.t. the T =15.6691 eV value: $\Delta E = E(T) - E(T = 15.6691 \text{ eV})$. Figure S4 (c) compares the total internal energies as provided by the approximate orbital-free DFT, and the reference MKS and PIMC sim-

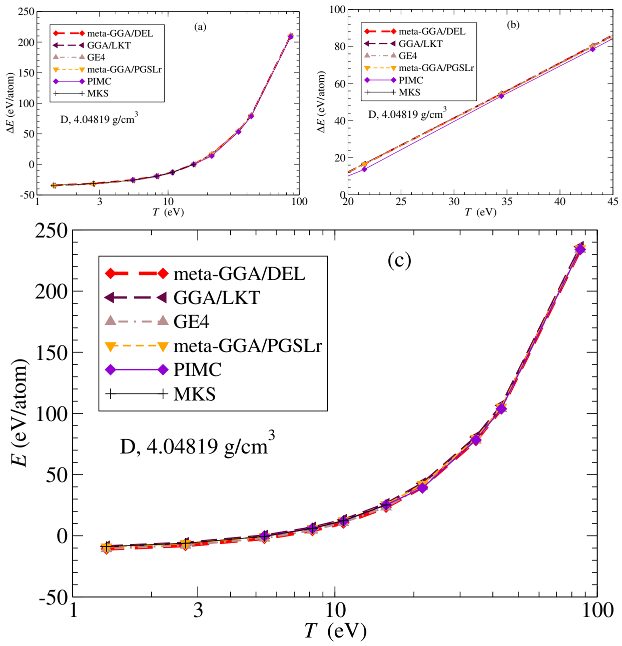

FIG. S4: **Panels (a) and (b):** The internal energy differences as functions of temperature from the density functional theory (DFT) molecular dynamics simulations of warm dense D at ρ = 4.04819 g/cm^3 (r_s = 1.1 bohr) with orbital-free density functionals, and the reference MKS and PIMC[22] data. Energy differences are calculated with respect to the total internal energy at T =15.6691 eV for each method: $\Delta E = E(T) - E(T = 15.6691 \text{ eV})$. T =15.6691 eV is the highest temperature available for the MKS data. **Panel (c):** The total internal energies as functions of temperature for the same set of calculations. All OF-DFT and MKS calculations use the local density approximation (LDA) ground-state XC[14].

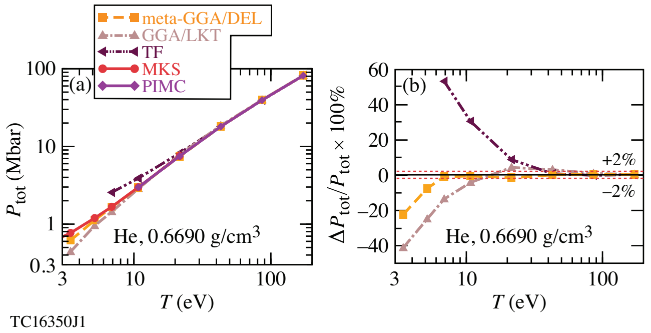

FIG. S5: (a) The total pressure from the orbital-free DFT AIMD simulations of warm dense He at $\rho = 0.6690$ g/cm^3 ($r_s = 2.0$ bohr) with meta-GGA/DEL [Eq. (15)] GGA/LKT[23] and TF functionals, and the reference MKS and PIMC[24] data. (b) The relative error of total pressure from OF-DFT simulations with respect to the combined MKS and PIMC data.

ulations. Energy differences and total energies from all calculations are indistinguishable on the scale of Figs. S4 (a) and (c). Figure S4 (b) shows that the orbital-free and MKS energies slightly disagree with the PIMC values in the range of temperatures between 20 eV and 45 eV. This disagreement can be explained by thermal XC effects missing in orbital-free and MKS calculations performed with the LDA ground-state XC. The Deuterium orbital-free and reference (MKS and PIMC) data including the total pressure, internal energy differences and total internal energies are tabulated in Table S5.

Figures S5-S7 show additional orbital-free and reference data for dense He. The total pressure from the OF-DFT AIMD simulations and the relative error with respect to the combined MKS and PIMC reference data along the 0.6690 g/cm^3 isochore, shown in Fig. S5 is discussed in the main text. Figures S6 and S7 compare between the orbital-free and MKS reference internal energy differences (panels (a)) and free energy differences (panels (b)) along two selected isochores. The internal and free energy differences are calculated w.r.t. the respective energy values at $T =$ 43.0886 eV for each method as shown in Figs. S6 and S7 captions. Panels (c) in Figs. S6 and S7 compare the total internal energies as provided by the approximate OF-DFT, and the reference MKS and PIMC simulations. In all cases we observe that the new meta-GGA/DEL orbital-free fucntional provides significant improvements of accuracy w.r.t. the MSK reference, as compared to the lower rung LDA/TF and GGA/LKT approximations. The Helium orbital-free and reference (MKS and PIMC) data including the total pressure, internal energy differences and total internal energy data along two isochores are tabulated in Table S6 and S7.

The total pressure, relative pressure differences, the internal and free energy differences, and the total internal energy for LiD along 3.2 g/cm^3 isochore are shown in Figs. S8 and S9. The meta-GGA/DEL and GGA/LKT orbital free total internal energy values are shifted by -5.561

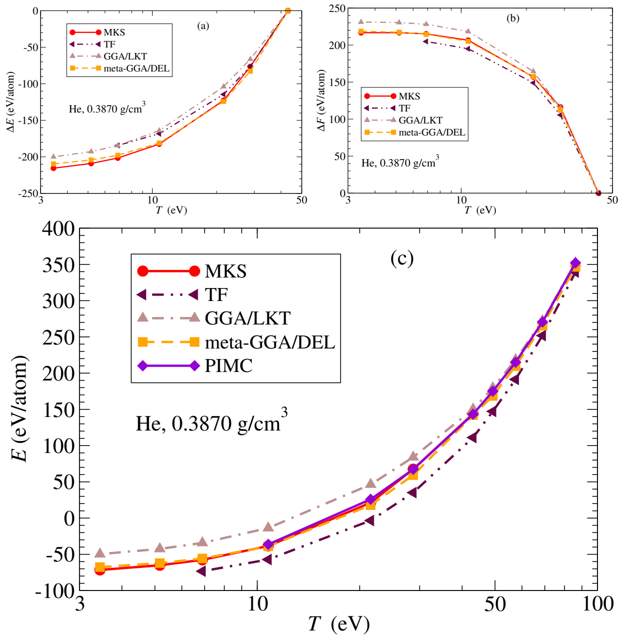

FIG. S6: The internal energy differences (**panel (a)**) and the free-energy differences (**panel (b)**) as functions of temperature from the DFT AIMD simulations of warm dense He at $\rho = 0.3870$ g/cm^3 ($r_s = 2.4$ bohr) with orbital-free density functionals, and the reference MKS and PIMC[24] data. Energy differences are calculated with respect to the total internal-/free- energy at T =43.0886 eV for each method: $\Delta E = E(T) - E(T = 43.0886$ eV) and $\Delta F = F(T) - F(T =$ 43.0886 eV) respectively. $T = 43.0886$ eV is the highest temperature available for the MKS data. **Panel (c):** The total internal energies as functions of temperature for the same set of calculations.

eV/atom and -30.433 eV/atom respectively to eliminate the pseudopotential differences and make the OF-DFT values compatible with the MKS data. Again, we observe substantial improvements of the meta-GGA/DEL results as compared to the GGA rung approximation for all reported quantities. The orbital-free and MKS data for the total pressure, internal energy differences and total internal energies are tabulated in Table S8.

Aluminum radial distribution functions at near-ambient and warm dense conditions obtained from orbital-free and MKS calculations are shown in Fig. S10. The newly developed meta-GGA/DEL orbital-free functional demonstrates much better agreement with the MKS reference as compared to the GGA/LKT orbital-free approximation.

Final remark: Nonlocal 2-point free-energy functionals are tied to the homogeneous electron gas (HEG) density response (Lindhard) function, that is a justified and reasonable approximation for (semi-) metallic systems with weakly inhomogeneous electron density. It is not surprising that existing non-local functionals provide excellent accuracy for metallic systems with weakly inhomogeneous electron density, such as warm dense D and Al. However, it is expected that such nonlocal functionals with enforced homogeneous gas response will

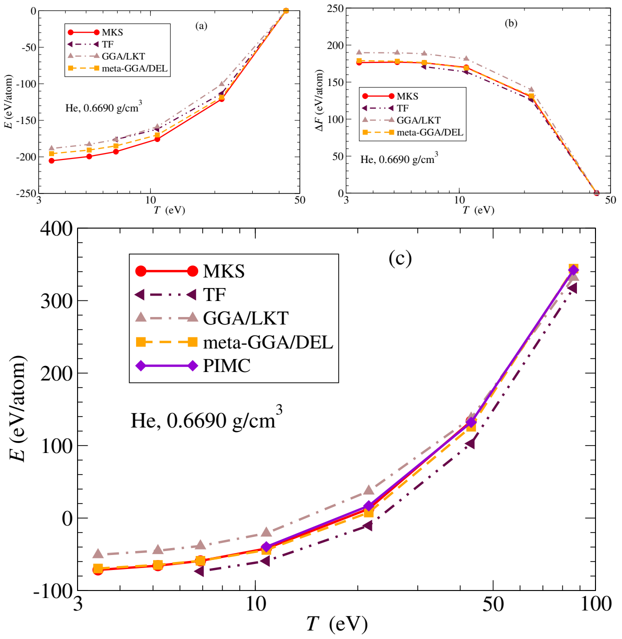

FIG. S7: The internal energy differences (**panel (a)**) and the free-energy differences (**panel (b)**) as functions of temperature from the density functional theory (DFT) molecular dynamics simulations of warm dense He at $\rho = 0.6690$ g/cm^3 ($r_s = 2.0$ bohr) with orbital-free density functionals, and the reference MKS and PIMC[24] data. Energy differences are calculated with respect to the total internal-/free- energy at T =43.0886 eV for each method: $\Delta E = E(T) - E(T = 43.0886$ eV) and $\Delta F = F(T) - F(T = 43.0886$ eV) respectively. T = 43.0886 eV is the highest temperature available for the MKS data. **Panel (c):** The total internal energies as functions of temperature for the same set of calculations. The meta-GGA/DEL and GGA/LKT orbital free values are shifted by -5.561 eV/atom and -30.433 eV/atom respectively to eliminate the pseudopotential differences and make them compatible with the MKS data.

not perform well when applied to insulators and systems with highly inhomogeneous electron densities (such as He, and LiD treated with hard all-electron pseudopotentials) when the HEG approximation fails.

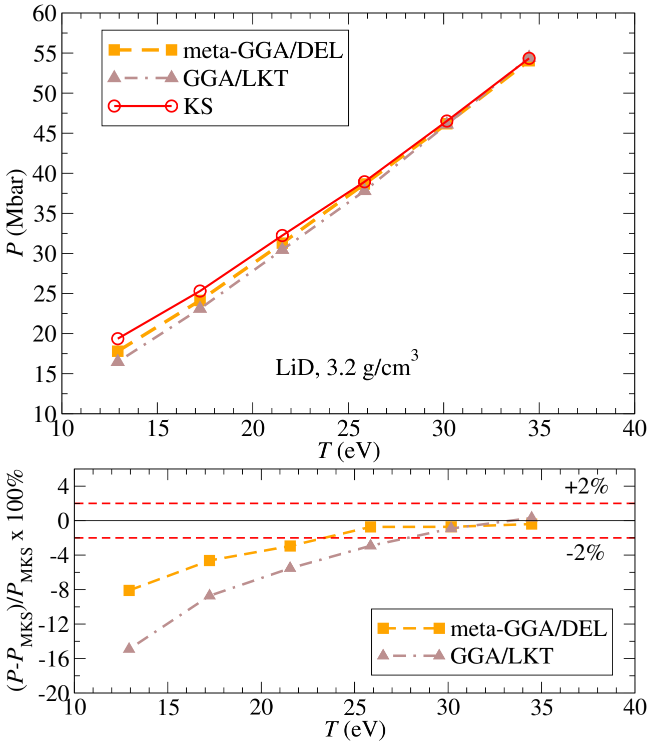

FIG. S8: The total pressure as function of temperature from the DFT AIMD simulations of warm dense LiD at ρ = 3.2 g/cm^3 with meta-GGA/DEL [Eq. (15)] and GGA/LKT [Ref.[23]] orbital-free functionals, and the reference MKS data.

V. ACKNOWLEDGMENTS

This material is based upon work supported by the Department of Energy National Nuclear Security Administration under Award Number DE-NA0004144, US National Science Foundation PHY Grant No. 2205521, the University of Rochester, and the New York State Energy Research and Development Authority.

This research used resources of the National Energy Research Scientific Computing Center, a DOE Office of Science User Facility supported by the Office of Science of the U.S. Department of Energy under Contract No. DE-AC02-05CH11231 using NERSC award FES-ERCAP0024726.

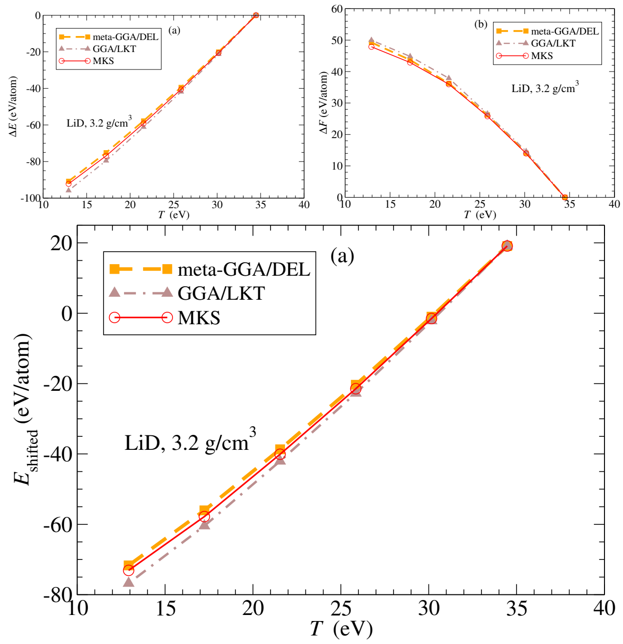

FIG. S9: The internal energy differences (panel (a)) and the free energy differences (panel (b)) as functions of temperature from the DFT AIMD simulations of warm dense LiD at $\rho = 3.2$ g/cm^3 with meta-GGA/DEL [Eq. (15)] and GGA/LKT [Ref.[23]] orbital-free functionals, and the reference MKS data. **Panel (c):** The total internal energy as a function of temperature for the same set of calculations. The meta-GGA/DEL and GGA/LKT orbital free values are shifted by -5.561 eV/atom and -30.433 eV/atom respectively to eliminate the pseudopotential differences and make them compatible with the MKS data.

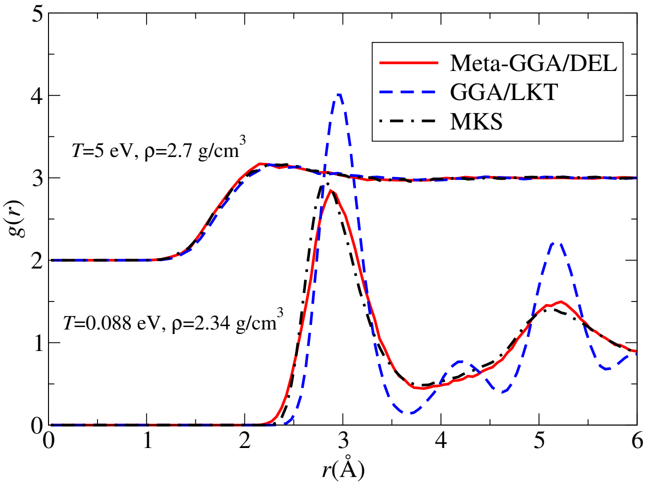

FIG. S10: Radial distribution function for Al at $\rho = 2.349$ g/cm^3 and $T = 0.088$ eV (lower curves) and at $\rho = 2.70$ g/cm^3 and $T = 5$ eV (upper curves shifted by 2) as predicted by the AIMD simulations with meta-GGA/DEL [Eq. (15)] and GGA/LKT[23] OF-DFT functionals, and the reference MKS data. All calculations were performed with the KDT16 thermal XC[21].

VI. TABLES

In this Section we present the coefficients of the analytic fits to $\widetilde{B}$, $\widetilde{C}$, $\widetilde{D}$, $\widetilde{E}$ as functions of the variable y. See details in Ref.[3] (Tables S1-S4). Values of the total pressure and internal energies for dense D, He and LiD along selected isochore(s) are tabulated in Tables S5-S8.

TABLE S1: Coefficients in fit to $\widetilde{B}(y)$.

coefficient	constraint	value
a_0	yes	3.0000000000000000
a_1	$-3/\sqrt{2\pi}$	-1.1968268412042982
a_2		427.3949714847699966
a_3		-170.1211444343163919
a_4		31.7020753506680002
a_5		3.3713851108273998
a_6		2.2529104734200001
a_7	yes	0.0000000000000000
a_8		0.0202417083225910
b_1	yes	0.0000000000000000
b_2	yes	0.0000000000000000
b_3		142.2807110810987865
b_4	yes	0.0000000000000000
b_5		0.5924932349226000
b_6		-18.0196644249469990
b_7		7.2322601129560002
b_8		0.1910870984626600
b_9		2.2522978973395000
b_{10}	$-2^{4/3}3^{-7/3}\pi^2 b_{12}$	-0.0387826345397392
b_{11}	yes	0.0000000000000000
b_{12}	a_8	0.0202417083225910

TABLE S2: Coefficients in fit to $\widetilde{C}(y)$.

coefficient	constraint	value
$a_{2.5}$		5.9265262369781002
a_1	$3^{5/3}2^{-5/3}$	1.9655560456566725
a_2		-0.5768378962095700
a_3		35.9130119576930014
a_4		41.1168867899709980
a_5		-40.3677476700629967
a_6		59.6804384544149968
a_7		-0.3211461169282900
a_8		4.2815226867198000
a_9		0.0030385200207883
a_{10}		0.1596522984577500
a_{11}	yes	0.0000000000000000
a_{12}		0.0056843727998872
b_1		26.5710993646139997
b_2		20.1172145257690005
b_3		17.6858602829550016
b_4		3.6467884940180002
b_5	$(a_{10} - C \times b_6) \times b_6/a_{12}$ [a]	0.1365086602125932
b_6	a_{12}	0.0056843727998872

[a] $C = 17 \times 2^{-5/3}3^{-7/3}\pi^2$.

TABLE S3: Coefficients in fit to $\widetilde{D}$(y).

coefficient	constraint	value
$a_{2.5}$		0.4524584047298800
a_1	$2^{1/3}3^{-1/3}$	0.8735804647362989
a_2		0.0300776040166210
a_3		14.3828916532949993
a_4		1.8670041583370001
a_5		37.9149736744980004
a_6		0.7589550686574100
a_7		16.9530731446740006
a_8		3.1373656916102002
a_9		0.0241382844920020
a_{10}		0.1538471708464500
a_{11}	yes	0.0000000000000000
a_{12}		0.0049093483855146
b_1		15.9188442750290005
b_2		29.1916070884210015
b_3		14.7377409947669999
b_4		3.1005334835656000
b_5	$(a_{10} - C \times b_6) \times b_6/a_{12}$ [a]	0.1178771827774314
b_6	a_{12}	0.0049093483855146

[a] $C = 413 \times 2^{-2/3}3^{-16/3}\pi^2$.

TABLE S4: Coefficients in fit to $\widetilde{E}(y)$.

coefficient	constraint	value
$a_{2.5}$	$3^{8/3}2^{-25/6}\pi^{-1/2}$	0.5881075583333214
a_1	yes	0.0
a_2	yes	0.0
a_3		-0.0132237512072000
a_4		0.5865252375234600
a_5		1.1120705517211000
a_6		2.2626091489173001
a_7		2.6723837550020000
a_8		0.3385116347002500
a_9		0.0038743130529412
a_{10}		0.0108166294882730
a_{11}	yes	0.0000000000000000
a_{12}		0.0003699371553596
b_1		2.8191769574094998
b_2		7.4555425143053000
b_3		2.5142144377484001
b_4		0.3944764252937600
b_5	$(a_{10} - C \times b_6) \times b_6/a_{12}$ [a]	0.0066524832876068
b_6	a_{12}	0.0003699371553596

[a] $C = 47 \times 2^{-5/3}3^{-7/3}\pi^2$.

TABLE S5: Orbital-free and MKS reference data for deuterium along the $\rho = 4.04819$ g/cm^3 isochore. PIMC data taken from Ref.[22] are tabulated as well. Energy differences along the isochore are calculated w.r.t. the T =181,825 K value, i.e. $\Delta E(T) = E(T) - E(T$=181,825K). All energy differences and energies are given in eV/atom units.

T (K)	Functional	P (Mbar)	ΔE	E
15,625	GGA/LKT	14.56	-34.84	-8.59
	GE4	15.88	-34.15	-10.56
	meta-GGA/PGSLr	14.56	-34.62	-9.12
	meta-GGA/DEL	15.55	-34.06	-10.72
	MKS	15.19	-34.08	-8.83
	PIMC	-	-	-
31.250	GGA/LKT	18.23	-31.96	-5.71
	GE4	19.52	-31.35	-7.77
	meta-GGA/PGSLr	18.22	-31.80	-6.30
	meta-GGA/DEL	19.23	-31.27	-7.93
	MKS	18.74	-31.39	-6.14
	PIMC	-	-	-
62,500	GGA/LKT	25.71	-26.05	0.20
	GE4	26.94	-25.55	-1.96
	meta-GGA/PGSLr	25.56	-26.05	-0.54
	meta-GGA/DEL	26.74	-25.46	-2.12
	MKS	26.05	-25.75	-0.50
	PIMC	26.2	-25.4	0.1
95,250	GGA/LKT	33.96	-19.46	6.78
	GE4	35.12	-19.05	4.53
	meta-GGA/PGSLr	33.88	-19.46	6.06
	meta-GGA/DEL	34.85	-19.06	4.28
	MKS	34.46	-19.19	6.07
	PIMC	34.4	-19.2	6.1
181,825	GGA/LKT	58.18	0.00	26.24
	GE4	58.71	0.00	23.58
	meta-GGA/PGSLr	58.20	0.00	25.50
	meta-GGA/DEL	58.53	0.00	23.34
	MKS	58.71	0.00	25.26
	PIMC	58.8	0.00	25.3
400,000	GGA/LKT	126.72	54.52	80.76
	GE4	126.10	54.60	78.19
	meta-GGA/PGSLr	126.37	54.07	79.57
	meta-GGA/DEL	126.02	54.45	77.79
	MKS	-	-	-
	PIMC	125.1	53.2	78.5
500,000	GGA/LKT	159.35	80.32	106.57
	GE4	158.38	80.31	103.89
	meta-GGA/PGSLr	158.81	79.65	105.16
	meta-GGA/DEL	158.40	80.38	103.72
	MKS	-	-	-
	PIMC	156.8	78.5	103.80
1,000,000	GGA/LKT	324.82	210.00	236.24
	GE4	323.57	210.46	234.04
	meta-GGA/PGSLr	323.91	209.21	234.71
	meta-GGA/DEL	323.60	210.69	234.03
	MKS	-	-	-
	PIMC	321.0	208.7	234.0

TABLE S6: Orbital-free and MKS reference data for helium along the $\rho = 0.3870$ g/cm^3 isochore. PIMC data taken from Ref.[24] are tabulated as well. Energy differences along the isochore are calculated w.r.t. the T =500,000 K value, i.e. $\Delta E(T) = E(T) - E(T$=500,000K). All energy differences and energies are given in eV/atom units.

T (K)	Functional	P (Mbar)	ΔE	E
40,000	TF	-	-	-
	GGA/LKT	0.260	-200.0	-49.75
	meta-GGA/DEL	0.319	-209.6	-67.60
	MKS	0.400	-215.5	-71.38
	PIMC	-	-	-
60,000	TF	-	-	-
	GGA/LKT	0.523	-192.8	-42.49
	meta-GGA/DEL	0.596	-204.1	-62.11
	MKS	0.649	-209.0	-64.90
	PIMC	-	-	-
80,000	TF	1.45	-184.4	-73.26
	GGA/LKT	0.832	-184.5	-34.26
	meta-GGA/DEL	0.910	-197.8	-55.75
	MKS	0.938	-201.6	-57.46
	PIMC	-	-	-
125,000	TF	2.24	-168.3	-57.14
	GGA/LKT	1.718	-164.0	-13.71
	meta-GGA/DEL	1.692	-181.0	-39.00
	MKS	1.728	-182.5	-38.37
	PIMC	1.723	-179.6	-36.28
250,000	TF	4.825	-114.5	-3.37
	GGA/LKT	4.735	-103.9	46.37
	meta-GGA/DEL	4.384	-124.2	17.87
	MKS	4.485	-122.7	21.44
	PIMC	4.457	-117.4	25.96
333,333	TF	6.721	-76.0	35.15
	GGA/LKT	6.810	-66.3	83.98
	meta-GGA/DEL	6.435	-82.8	59.27
	MKS	6.54	-76.3	67.62
	PIMC	6.514	-76.0	67.32
500,000	TF	10.694	0.0	111.15
	GGA/LKT	10.710	0.0	150.27
	meta-GGA/DEL	10.710	0.0	142.03
	MKS	10.69	0.0	143.95
	PIMC	10.677	0.0	143.35
571,428	TF	12.442	36.0	147.18
	GGA/LKT	12.523	29.4	179.69
	meta-GGA/DEL	12.523	31.9	173.89
	MKS	-	-	-
	PIMC	12.499	31.7	175.02
666,667	TF	14.774	80.2	191.37
	GGA/LKT	14.877	68.2	218.45
	meta-GGA/DEL	14.877	73.1	215.17
	MKS	-	-	-
	PIMC	14.842	71.7	215.01
800,000	TF	18.052	140.6	251.78
	GGA/LKT	18.198	121.9	272.17
	meta-GGA/DEL	18.198	129.0	271.05
	MKS	-	-	-
	PIMC	18.155	127.2	270.59
1,000,000	TF	23.003	228.3	339.43
	GGA/LKT	23.120	201.8	352.02
	meta-GGA/DEL	23.120	210.0	352.05
	MKS	-	-	-
	PIMC	23.084	208.8	352.12

TABLE S7: Orbital-free and MKS reference data for helium along the $\rho = 0.6690$ g/cm^3 isochore. PIMC data taken from Ref.[24] are tabulated as well. Energy differences along the isochore are calculated w.r.t. the T =500,000 K value, i.e. $\Delta E(T) = E(T) - E(T$=500,000K). All energy differences and energies are given in eV/atom units.

T (K)	Functional	P (Mbar)	ΔE	E
40,000	TF	-	-	-
	GGA/LKT	0.452	-188.5	-50.66
	meta-GGA/DEL	0.597	-195.6	-69.55
	MKS	0.770	-205.3	-71.53
	PIMC	-	-	-
60,000	TF	-	-	-
	GGA/LKT	0.90	-182.9	-45.01
	meta-GGA/DEL	1.11	-190.6	-64.62
	MKS	1.20	-199.4	-65.67
	PIMC	-	-	-
80,000	TF	2.56	-176.3	-73.37
	GGA/LKT	1.442	-176.3	-38.42
	meta-GGA/DEL	1.653	-185.0	-58.95
	MKS	1.671	-192.9	-59.09
	PIMC	-	-	-
125,000	TF	3.89	-162.1	-59.23
	GGA/LKT	2.856	-158.7	-20.90
	meta-GGA/DEL	2.966	-170.0	-44.02
	MKS	2.985	-175.7	-41.98
	PIMC	2.974	-171.8	-39.68
250,000	TF	8.189	-113.2	-10.32
	GGA/LKT	7.854	-100.7	37.13
	meta-GGA/DEL	7.411	-118.4	7.63
	MKS	7.537	-121.0	12.81
	PIMC	7.459	-115.1	16.94
500,000	TF	18.127	0.0	102.89
	GGA/LKT	18.545	0.0	137.83
	meta-GGA/DEL	18.006	0.0	126.02
	MKS	18.11	0.0	133.76
	PIMC	18.009	0.0	132.08
1,000,000	TF	39.307	221.4	324.25
	GGA/LKT	39.718	194.2	332.07
	meta-GGA/DEL	39.523	218.1	344.14
	MKS	-	-	-
	PIMC	39.412	210.2	342.31

TABLE S8: Orbital-free and MKS reference data for LiD along the $\rho = 3.2$ g/cm^3 isochore. All energies are shifted to zero at T =400,000 K. Energy differences along the isochore are calculated w.r.t. the T =400,000 K value, i.e. $\Delta E(T) = E(T) - E(T$=400,000K). All energy differences and energies are given in eV/atom units.

T (K)	Functional	P (Mbar)	ΔE	E	Eshifted
150,000	GGA/LKT	16.48	-95.88	-46.345	-76.778
	meta-GGA/DEL	17.80	-90.77	-66.111	-71.672
	MKS	19.36	-92.20	-73.096	-73.096
200,000	GGA/LKT	23.10	-79.54	-30.012	-60.445
	meta-GGA/DEL	24.14	-75.13	-50.469	-56.030
	MKS	25.31	-76.90	-57.799	-57.799
250,000	GGA/LKT	30.45	-61.14	-11.607	-42.040
	meta-GGA/DEL	31.28	-57.77	-33.113	-38.674
	MKS	32.23	-59.18	-40.084	-40.084
300,000	GGA/LKT	37.82	-41.83	7.698	-22.735
	meta-GGA/DEL	38.66	-39.42	-14.763	-20.324
	MKS	38.95	-40.55	-21.450	-21.450
350,000	GGA/LKT	46.10	-21.25	28.283	-2.150
	meta-GGA/DEL	46.19	-20.06	4.599	-0.962
	MKS	46.52	-20.61	-1.510	-1.510
400,000	GGA/LKT	54.43	0.00	49.532	19.099
	meta-GGA/DEL	54.05	0.00	24.660	19.099
	MKS	54.28	0.00	19.099	19.099

(1) Karasiev, V. V.; Sjostrom, T.; Trickey, S. B. Generalized-gradient-approximation noninteracting free-energy functionals for orbital-free density functional calculations. *Phys. Rev. B* **2012**, *86*, 115101 (2012).

(2) Perrot, F. Gradient correction to the statistical electronic free energy at nonzero temperatures: application to equation-of-state calculations. *Phys. Rev. A* **1979**. *20*, 586-594.

(3) Karasiev, V. V.; Chakraborty, D.; Trickey, S.B. Improved analytical representation of combinations of Fermi–Dirac integrals for finite-temperature density functional calculations. *Comput. Phys. Commun.* **2015**, *192*, 114-123.

(4) Geldart, D. J. M.; Sommer, E. Fourth order gradient corrections to the free energy of noninteracting many-electron systems at finite temperature. *Phys. Lett. A* **1985**, *108*, 103-104.

(5) Geldart, D. J. W.; Sommer, E. Fourth-order gradient contributions in extended Thomas-Fermi theory for non-interacting fermions at finite temperature. *Phys. Rev. B* **1985**, *32*, 7694-7702.

(6) Bartel, J.; Brack, M.; Durand, M. Extended Thomas-Fermi theory at finite temperature. *Nucl. Phys. A* **1985**, *445*, 263-303.

(7) VKarasiev, V. V.; Sjostrom, T.; Trickey, S. B. Finite-temperature orbital-free DFT molecular dynamics: Coupling PROFESS and QUANTUM ESPRESSO. *Comput. Phys. Commun.* **2014**, *185*, 3240-3249.

(8) Ho, G. S.; Lignères V. L.; Carter, E. A. Introducing PROFESS: A new program for orbital-free density functional theory calculations. *Comput. Phys. Commun.* **(**2008), *179*, 839-854.

(9) Hung, L.; Huang, C.; Shin, I.; Ho, G. S.; Lignères, V. L.; Carter, E. A. Introducing PROFESS 2.0: A parallelized, fully linear scaling program for orbital-free density functional theory calculations. *Comput. Phys. Commun.* **2010**, *181*, 2208-2209.

(10) Chen, M.; Xia, J.; Huang, C.; Dieterich, J. M.; Hung, L.; Shin, I.; Carter, E. A. Introducing PROFESS 3.0: An advanced program for orbital-free density functional theory molecular dynamics simulations. *Comput. Phys. Commun.* **2015**, *190*, 228-230.

(11) Andersen, H. C. Molecular dynamics simulations at constant pressure and/or temperature. *J. Chem. Phys.* **1980**, *72*, 2384-2393.

(12) Berendsen, H. J. C.; Postma, J. P. M.; van Gunsteren, W. F.; DiNola, A.; Haak, J. R. Molecular dynamics with coupling to an external bath. *J. Chem. Phys.* **1984**, *81*, 3684-3690.

(13) Kresse G.; Furthmüller, J. Efficient iterative schemes for ab initio total-energy calculations using a plane-wave basis set", Phys. Rev. B **54**, 11169-11186.

(14) Perdew, J. P.; Zunger, A. Self-interaction correction to density-functional approximations for many-electron systems. *Phys. Rev. B* **1981**, *23*, 5048-5079.

(15) Sjostrom, T.; Daligault J. Fast and accurate quantum molecular dynamics of dense plasmas across temperature regimes. *Phys. Rev. Lett.* **2014**, *113*, 155006(1-5).

(16) Hartwigsen, C.; Goedecker, S.; Hutter, J. Relativistic separable dual-space Gaussian pseudopotentials from H to Rn. *Phys. Rev. B* **1998**, *58*, 3641-3662.

(17) Baldcreschi, A. Mean-value point in the Brillouin zone. *Phys. Rev. B* **1973**, *7*, 5212-5214.

(18) Karasiev, V. V.; Mihaylov, D. I.; Hu, S. X. Meta-GGA exchange-correlation free energy density functional to increase the accuracy of warm dense matter simulations. *Phys. Rev. B* **2022**, *105*, L081109(1-7).

(19) Heine, V.; Abarenkov, I. V. A new method for the electronic structure of metals. *Phil. Mag.* **1964**, *9*, 451-465.

(20) Goodwin, L.; Needs, R. J.; Heine, V. A pseudopotential total energy study of impurity-promoted intergranular embrittlement. *J. Phys.: Condens. Matter* **1990**, *2*, 351-365.

(21) Karasiev, V. V.; Dufty, J.; Trickey, S. B. Non-empirical semi-local free-energy density functional for matter under extreme conditions. *Phys. Rev. Lett.* **2018**, *120*, 076401(1-7).

(22) Hu, S. X.; Militzer, B.; Goncharov, V. N.; Skupsky, S. First-principles equation-of-state table of deuterium for inertial confinement fusion applications. *Phys. Rev. B* **2011** *84* 224109(1-18) (2011).

(23) Luo, K.; Karasiev, V. V.; Trickey, S. B. Towards accurate orbital-free simulations: a generalized gradient approximation for the noninteracting free energy density functional. *Phys. Rev. B* **2020**, *101*, 075116(1-9).

(24) Militzer, B. Path integral Monte Carlo and density functional molecular dynamics simulations of hot, dense helium. *Phys. Rev. B* **2009**, *79*, 155105(1-18).
